# Artificial intelligence-based diagnostic model construction and quantitative analysis of retinal vein occlusion lesions using color fundus photographs

**DOI:** 10.3389/fcell.2026.1888364

**Published:** 2026-09-29

**Authors:** Wenjuan Wu, Li Sun, Xingye Wang, Yan Liao, Jinrong Liu, Liwen He, Juan Liu, Jie Wang, Yilin Zhong, Hongyun Wu, Dennis S. C. Lam, Zhe Xu

**Affiliations:** 1 Department of Ophthalmology, Ganzhou Hospital-Nanfang Hospital,Southern Medical University (Ganzhou People’s Hospital), Ganzhou, Jiangxi, China; 2 The Affiliated Ganzhou Hospital, Jiangxi Medical College, Nanchang University, Ganzhou, Jiangxi, China; 3 Ganzhou Key Laboratory of Ocular Trauma, Ganzhou People’s Hospital, Ganzhou, Jiangxi, China; 4 Department of Ophthalmology, Zhujiang Hospital of Southern Medical University, Guangzhou, Guangdong, China; 5 Department of Ophthalmology, C-MER (Guangzhou) Dennis Lam Eye Hospital, Guangzhou, Guangdong, China; 6 EVision Technology (Beijing) Co., LTD, Beijing, China; 7 Health Management Center, The Third Affiliated Hospital of Southern Medical University, Guangzhou, Guangdong, China; 8 The Primasia International Eye Research Institute (PIERI), School of Medicine, The Chinese University of Hong Kong (Shenzhen), Shenzhen, Guangdong, China; 9 The C-MER Dennis Lam & Partners Eye Center, C-MER International Eye Care Group, Hong Kong, Hong Kong SAR, China

**Keywords:** retinal vein occlusion, color fundus photographs, automated segmentation, quantitative assessment, artificial intelligence

## Abstract

Retinal vein occlusion (RVO) is the second most common retinal vascular disease worldwide, affecting over 7 million people in China. Currently, the clinical diagnosis and severity assessment of RVO largely rely on the subjective judgment of physicians, leading to high rates of missed diagnosis at an early stage, insufficient quantitative assessment, and poor consistency during follow-up. Most existing RVO-related AI models only support classification diagnosis and lack multi-dimensional quantitative assessment capabilities. This study evaluated an existing AI system (EVisionAI) for automatic segmentation and quantitative analysis of RVO lesions based on color fundus photographs, using a dataset of 106 RVO eyes and 60 normal control eyes. The system can simultaneously extract three core indicators: lesion characteristics, vascular morphology, and fundus tessellation density. The results showed that the Dice coefficients of the AI for the segmentation of hemorrhage, hard exudate, and cotton-wool spots were all above 0.78. After adjusting for demographic factors and systemic vascular risk factors in a multivariable GEE model, there were significant associations between the total area of hemorrhage, vascular fractal dimension, and vascular density for RVO (all P < 0.05), suggesting these metrics as promising objective biomarkers for RVO evaluation. A diagnostic model based on these multidimensional indicators achieved a cross-validated area under the curve (AUC) of 0.951 (SD: 0.018) for distinguishing RVO eyes from healthy controls. In summary, the proposed AI system enables precise, standardized, and reproducible multi-parametric quantitative evaluation of RVO. However, given the single-center cross-sectional design and the absence of external validation and disease controls, these findings are preliminary. Prospective multi-center studies with external validation are required to confirm its clinical utility.

## 1 Introduction

Retinal vein occlusion (RVO) is a common retinal vascular disease caused by obstruction of retinal venous outflow, with a population prevalence of 0.7%–1.6%. Its main complications include macular edema, neovascular glaucoma, and vitreous hemorrhage, and it is one of the main causes of visual impairment in the working-age population (Romano et al., 2023; Ip and Hendrick, 2018; Chen et al., 2021). According to the location of the obstruction, it can be classified into branch retinal vein occlusion (BRVO) and central retinal vein occlusion (CRVO), and the risk characteristics, prognosis, and treatment plans of the two types of lesions are significantly different (Zhang et al., 2022; Tang et al., 2022; Yu, 2025).

Currently, the diagnosis of RVO in clinical practice mainly relies on color fundus photography as the core imaging basis (Lendzioszek et al., 2024). It mainly depends on the subjective interpretation of characteristic fundus changes such as tortuous and dilated veins, hemorrhage along the vascular course, cotton-wool spots, and hard exudates by physicians. This can only achieve qualitative diagnosis and cannot complete the precise segmentation of lesions and objective quantitative assessment of severity (Heitmar et al., 2015). This situation leads to three core clinical pain points: First, early RVO lesions are concealed, and the missed diagnosis rate by non-ophthalmic specialists in primary care can reach over 30%, causing many patients to miss the best intervention time. Second, there is a lack of a unified quantitative standard for severity assessment, and the consistency of assessment among different physicians is only 0.6 to 0.7, which is not conducive to the precise formulation of individualized treatment plans (Castro et al., 2023). Third, it is difficult to precisely quantify the changes in lesions during follow-up, making it impossible to objectively evaluate the response to treatments such as anti-vascular endothelial growth factor (anti-VEGF), and timely adjustment of treatment plans is difficult. Therefore, the development of an automated analysis method that can objectively and precisely assess RVO is of great clinical value for improving diagnostic accuracy, guiding individualized treatment, and optimizing the entire disease management process.

In recent years, artificial intelligence has made remarkable progress in the field of medical image analysis, and its application in ophthalmology has also shown great potential. Algorithms based on deep learning (DL) and machine learning (ML) can efficiently and automatically process fundus images, achieving precise localization, segmentation, and quantitative analysis of lesion areas (Zapata et al., 2020; Lim et al., 2024; Wu J. et al., 2024; Yang et al., 2023; Yuan et al., 2025). A large number of studies have confirmed the value of artificial intelligence-assisted diagnostic systems in the screening, diagnosis, and prediction of common retinal diseases such as diabetic retinopathy (DR), age-related macular degeneration (AMD), and glaucoma. Relevant technical achievements have begun to be applied in preliminary clinical screening (Feng et al., 2024; Rojas-Carabali et al., 2024; Dong et al., 2021; Grzybowski et al., 2024; Chen et al., 2024). This study developed an automatic segmentation and quantitative assessment system for RVO lesions based on retinal fundus images. The system utilizes advanced image processing algorithms and leverages characteristic imaging manifestations to achieve automatic segmentation and quantitative analysis of RVO-related lesions (such as hemorrhage and exudation) as well as retinal vascular structure and morphology. It is expected that this system will provide objective and accurate quantitative data support for the diagnosis, treatment, and prognosis assessment of RVO.

## 2 Materials and methods

### 2.1 Study design and participants

This cross-sectional study aimed to develop and validate an automated segmentation and quantitative assessment system for RVO lesions based on fundus images. The study protocol was reviewed and approved by the Institutional Ethics Committee of Ganzhou People’s Hospital (Approval No. TY-ZKY2022-101-01), and written informed consent was obtained from all participants prior to enrollment. The study was conducted in strict accordance with the ethical principles of the Declaration of Helsinki.

The RVO group comprised eyes diagnosed with either branch or central retinal vein occlusion by consensus of at least two senior ophthalmologists based on characteristic fundus findings including retinal vein tortuosity and dilation, multifocal flame-shaped or dot/blot hemorrhages along the vessel arcades, cotton-wool spots, or macular edema. The control group consisted of either healthy volunteers undergoing routine ophthalmic examination or patients with simple refractive errors, all of whom demonstrated no evidence of retinal vascular diseases, macular disorders, glaucoma, or other significant fundus pathologies upon comprehensive ophthalmic evaluation. Participants from both groups were excluded in cases of significant media opacity, such as corneal leucoma, severe cataract, or vitreous hemorrhage, that compromised fundus image quality, as well as in the presence of other confounding fundus diseases including diabetic retinopathy, age-related macular degeneration, high myopic retinopathy (with fundus tessellation severity ≥ Grade C), retinal artery occlusion, or uveitis. A history of intraocular surgery except for uncomplicated cataract surgery performed more than 3 months prior with stable postoperative condition also served as grounds for exclusion. A total of 20 eyes (12 from potential RVO subjects, eight from potential controls) were excluded due to poor image quality, leaving 166 eyes for final analysis. The two senior ophthalmologists reached a consensus diagnosis for all included RVO cases. In cases of initial disagreement, a discussion was held until a consensus was achieved, ensuring a unified and reliable clinical reference standard. Ultimately, 106 eyes were assigned to the RVO group and 60 eyes to the control group.

### 2.2 Data acquisition and ophthalmic imaging

All participants underwent systematic ophthalmic examinations, including measurement of best-corrected visual acuity (BCVA), intraocular pressure (IOP) using non-contact tonometry, slit-lamp anterior segment examination, and fundus imaging. The primary imaging data consisted of color fundus photographs acquired using a standardized device (Zeiss CLARUS 500). Ultra-widefield (UWF) images centered on the macula were obtained. To ensure image quality met the requirements for subsequent automated analysis, two independent evaluators, masked to the clinical classifications, screened all images. Those with significant media opacities, defocus, or over-/under-exposure were excluded.

### 2.3 Automated segmentation and quantitative analysis system

The core analytical tool used in this study is the EVisionAI artificial intelligence system (developed by EVision Technology (Beijing) Co. Ltd.), which is designed for automated lesion segmentation and quantitative analysis of retinal fundus images. This study evaluated this existing commercial AI system rather than developing a new model from scratch. The system is built upon a deep learning architecture with a human vision simulation mechanism, integrating computer vision and deep learning techniques for automated retinal feature segmentation and quantification. For vessel and peripapillary atrophy segmentation, the system employs a ResNet101-UNet segmentation network (He et al., 2024); for optic disc detection, a Single Shot Detection model with ResNet-50 as the backbone network is used (Liu et al., 2023). The model was trained on large-scale, expert-annotated datasets comprising 40,600 fundus images from Shanghai community residents, 2,981 patients from Tianjin Eye Hospital, and 12,067 photographs from 5,320 patients at Beijing Tongren Hospital (He et al., 2024; Long et al., 2022; He et al., 2023). All images were acquired using standardized fundus cameras, with annotations performed by board-certified ophthalmologists and adjudicated by senior clinicians (He et al., 2024; He et al., 2023). The model was trained on training samples, evaluated on a verification set, and final performance was assessed on an independent test set (He et al., 2024). The analytical pipeline comprises several sequential stages: image preprocessing and enhancement, lesion segmentation via the deep learning model, post-processing refinement of segmentation outputs, and final extraction of comprehensive quantitative parameters. Based on the segmentation results, the system automatically calculates key metrics, including lesion-specific indicators (number, total area, and maximum area of hemorrhages, hard exudates, and cotton-wool spots across retinal subregions), vascular morphological parameters (fractal dimension, vessel diameter, vessel mean curvature, vessel density, and vessel length derived from skeletonization and diameter measurement algorithms) (Zapata et al., 2020; Jin et al., 2022), and fundus tessellation density (FTD, computed using a texture analysis algorithm in regions such as the peripapillary and macular areas) (Chen et al., 2024; Long et al., 2022).

### 2.4 Quantitative parameter definitions and calculation

To provide a comprehensive characterization of retinal alterations in RVO, the EVisionAI system automatically computed a panel of quantitative metrics spanning vascular morphology, lesion burden, and fundus tessellation. The definitions and calculation methods for each parameter are detailed below.

Region of Interest (ROI) encompassed the entire valid fundus region after excluding non-retinal areas (e.g., dark corners, optic disc artifacts, and over-/under-exposed regions). This whole-image ROI was applied to cumulative metrics, including total lesion counts, total lesion areas, vessel length, and overall FTD. For zonal analyses, the ROI was further partitioned into regions centered on the optic disc or the macula. Specifically, peripapillary zones and macular-centered concentric rings (e.g., R1, R2, R3) were defined to compute zonal statistics for vascular morphological parameters, while quadrantic divisions (superior, inferior, nasal, temporal) were applied for regional sub-analyses (e.g., Circle Vessel Diameter Z3).

Fractal Dimension is a unitless metric that captures the geometric complexity, irregularity, and self-similarity of the retinal vascular network. In fractal geometry, greater self-similarity across spatial scales, where vascular patterns appear topologically similar at different magnifications, results in Fractal Dimension values approaching 2, reflecting increasingly intricate branching architecture. In contrast, when vascular branching becomes sparse or structurally simplified, Fractal Dimension values tend toward 1 (Cheung et al., 2012). To compute Fractal Dimension, the binarized vessel skeletons generated by the segmentation network were overlaid with a grid of ε-length cells, and the number of grid cells containing vessel pixels, N(ε), was enumerated. Fractal Dimension was subsequently derived using the following formula:

$$\text{Fractal Dimension}=\lim\left(\varepsilon \to 0\right)\log N\left(\varepsilon \right)/\log\left(1/\varepsilon \right)$$

tRatio Vessel (Vessel Density) represents the percentage of vascular pixels within the region of interest (ROI), reflecting retinal blood flow perfusion level. Following vascular segmentation, the resulting vessel probability maps were converted into binary masks. Vessel density was calculated as:

$$\text{tRatio Vessel}=\left(\text{Vessel Pixels}/\text{ROI Pixels}\right)\times 100\%$$

Vessel Mean Curvature quantifies the average angular deviations along the vascular centerlines. In practice, the centerline of each extracted vessel was reduced to a sequence of interconnected line segments, and the mean angular change between successive segments was taken as the Vessel Mean Curvature (Badawi et al., 2022). Curvature per segment was calculated as the ratio of the actual path length to the Euclidean distance between the segment endpoints:

$$\text{Curvature per segment}=\text{Actual Length}/\text{Straight}-\text{line Distance};\text{ Vessel Mean Curvature}=\Sigma \left(\text{Curvature}\_i\right)/N$$

Vessel Diameter is defined as the average distance across the vessel lumen, measured along lines perpendicular to the tangent direction at sampled points on the vascular centerline. Specifically, the vascular region was first delineated through image segmentation to extract both the centerline and the vessel boundaries. Along each centerline, sampling points were selected at 10-pixel intervals. At each sampling point, a line was constructed perpendicular to the local tangent direction, and the intersection distance with the vessel wall boundaries was measured. The mean of all such intersection distances across all sampled points within the entire fundus region was taken as the mean vessel diameter. The final diameter value in micrometers was derived by multiplying the pixel-based measurement by the pixel resolution: $\text{Vessel Diameter }\left(\mu m\right)=\text{Vessel Diameter }\left(\text{pixels}\right)\times \text{Pixel Resolution }\left(\mu m/\text{pixel}\right)$ . Arteries and veins were not analyzed separately in the current study; the reported diameter represents the combined average of all detectable vessels.

Vessel Length denotes the cumulative sum of centerline lengths of all extracted vessels within the entire fundus region. Following vessel segmentation and skeletonization, the vascular centerlines were refined to single-pixel width. Sub-pixel fitting was subsequently applied to generate a continuous sequence of centerline points with sub-pixel precision. The Euclidean distance between all adjacent points was calculated and summed to obtain the total vessel length (in micrometers) (Martinez-Perez et al., 2002). The large and variable Vessel Length values observed in RVO eyes reflect the algorithmic interpretation of highly tortuous and dilated venous segments as longer continuous paths, serving as a quantitative morphological descriptor of these pathological changes rather than an absolute biological measure.

FTD represents the proportion of the fundus tessellation area within the captured fundus region on color fundus photographs, as identified by the image segmentation model. The segmentation algorithm first delineated both the tessellation regions and the total fundus region (He et al., 2023). FTD was then calculated as the ratio of pixel counts between the tessellation area and the total fundus area:

$$\text{FTD}=\left(\text{Tessellation Pixels}/\text{Total Fundus Pixels}\right)\times 100\%$$

### 2.5 Statistical analysis

Data analysis was conducted using SPSS software, version 27.0 (IBM Corp., Armonk, NY, United States of America) and R software (version 4.2.0). Given that both eyes from the same participant were included, all analyses were performed using methods that explicitly account for within-subject inter-eye correlation. Continuous variables are expressed as mean ± standard deviation. For intergroup comparisons, linear mixed-effects models with participant ID as a random effect were used for normally distributed data; for variables that did not meet the assumption of normality, data were transformed or nonparametric tests within the mixed model framework were applied. The strength and direction of associations between retinal microvascular parameters and RVO status were evaluated using generalized estimating equations (GEE) with an exchangeable correlation structure. To identify independent correlates of RVO and adjust for potential confounders, a multivariable logistic regression model was constructed using GEE. The model included age, sex, eye side, Fractal Dimension, tRatio Vessel, hypertension, and dyslipidemia, which were significantly imbalanced between groups in baseline comparisons, to account for their potential confounding effects. Variables were selected based on clinical relevance and baseline imbalances; no significant multicollinearity was detected (variance inflation factor <5 for all included variables). Statistical significance was defined as a two-tailed p-value less than 0.05. All confidence intervals reported are at the 95% level. To evaluate the diagnostic performance of the AI system and mitigate potential overfitting, we performed a 5-fold cross-validation on the entire dataset (166 eyes). The mean accuracy, sensitivity, specificity, and area under the curve (AUC) across the five folds were reported.

## 3 Results

### 3.1 Study participants and data distribution

A total of 166 eyes were included in this study. Based on clinical evaluation, participants were divided into an RVO group (n = 106) and a healthy control group (n = 60). The baseline demographic characteristics of the two groups are presented in Table 1. The RVO group was significantly older than the control group (57.49 ± 12.15 years vs. 52.13 ± 11.90 years, P < 0.001), which is consistent with the higher burden of systemic vascular risk factors observed in this cohort. No statistically significant differences were observed in sex distribution (male: 58.49% vs. 51.67%, *P* = 0.410) or BMI (23.61 ± 3.62 vs. 24.37 ± 3.70 kg/m^2^, *P* = 0.176). Further analysis of the RVO group revealed that BRVO was the predominant type, comprising 74 cases (69.81%), while CRVO accounted for 32 cases (30.19%). Statistical analysis confirmed no significant predilection for RVO occurrence between left and right eyes. Substantial imbalances were noted in systemic vascular risk factors: as shown in Table 1, smoking history (0% vs. 20.8%, *P* < 0.001), hypertension (20.0% vs. 81.1%, *P* < 0.001), and dyslipidemia (25.0% vs. 57.5%, *P* < 0.001) were significantly more common in the RVO group. Regarding lipid profiles, total cholesterol (5.18 ± 1.07 vs. 4.78 ± 0.85 mmol/L, *P* < 0.001), triglycerides (1.72 ± 0.79 vs. 1.48 ± 0.70 mmol/L, *P* < 0.001), and LDL cholesterol (3.10 ± 0.87 vs. 2.49 ± 0.75 mmol/L, *P* < 0.001) were significantly higher in the RVO group compared to controls, while HDL cholesterol showed no significant difference (1.36 ± 0.33 vs. 1.35 ± 0.32 mmol/L, *P* = 0.602). These imbalances were appropriately adjusted for in the subsequent multivariable GEE analyses.

**TABLE 1:** Baseline participant characteristics and clinic assessment.

Characteristics	Control (n = 60)	RVO (n = 106)	*P* value
Age (mean/SD)	52.13 ± 11.90	57.49 ± 12.15	*P* < 0.001
Sex (male/female)	31/29	62/44	*P* = 0.410
BMI (mean/SD), kg/m^2^	23.61 ± 3.62	24.37 ± 3.70	*P* = 0.176
Smoking	0	22 (20.8%)	*P* < 0.001
Hypertension, n (%)	12 (20.0%)	86 (81.1%)	*P* < 0.001
Total cholesterol (mmol/L)	4.78 ± 0.85	5.18 ± 1.07	*P* < 0.001
Triglycerides (mmol/L)	1.48 ± 0.70	1.72 ± 0.79	*P* < 0.001
HDL cholesterol (mmol/L)	1.35 ± 0.32	1.36 ± 0.33	*P* = 0.602
LDL cholesterol (mmol/L)	2.49 ± 0.75	3.10 ± 0.87	*P* < 0.001
Dyslipidemia, n (%)	15 (25.0%)	61 (57.5%)	*P* < 0.001

Abbreviations: BMI, body mass index; HDL, high-density lipoprotein; LDL, low-density lipoprotein.

### 3.2 Quantitative analysis of retinal vascular structure and morphology in RVO

The segmentation performance of the AI system was validated against manual annotations by two independent graders on a randomly selected subset of 60 images. The average Dice similarity coefficients were 0.85 for hemorrhages, 0.78 for hard exudates, and 0.82 for cotton-wool spots, indicating good agreement. The inter-grader Dice coefficient was 0.82.

Utilizing automated image quantitative analysis technology (Figures 1, 2), we systematically quantified and compared retinal vascular morphology and lesion characteristics between the RVO group and the healthy control group. Quantitative results (Table 2) revealed significant alterations in multiple imaging dimensions in RVO-affected eyes compared to controls (all metrics *P* < 0.05). Lesion-specific metrics in the RVO group, including the number of hemorrhage foci, total hemorrhage area, and maximum hemorrhage area, were significantly elevated. Concurrently, the fractal dimension of the vasculature was also significantly higher in RVO eyes. In contrast, markers indicative of impaired perfusion, namely, mean vessel diameter and vessel density, were significantly reduced in the RVO group. Notably, the calculated “Vessel Length” parameter showed a large increase in the RVO group. This metric is derived from the skeletonized vascular network, and its marked elevation in RVO likely reflects the algorithmic interpretation of highly tortuous and dilated venous segments as longer continuous paths, serving as a quantitative morphological descriptor of these pathological changes rather than an absolute biological measure. This multi-parameter analysis demonstrates significant differences in lesion burden, retinal vascular morphology, and density between RVO and healthy eyes, suggesting their potential utility as imaging biomarkers for characterizing RVO pathophysiology and distinguishing disease status.

**FIGURE 1:**
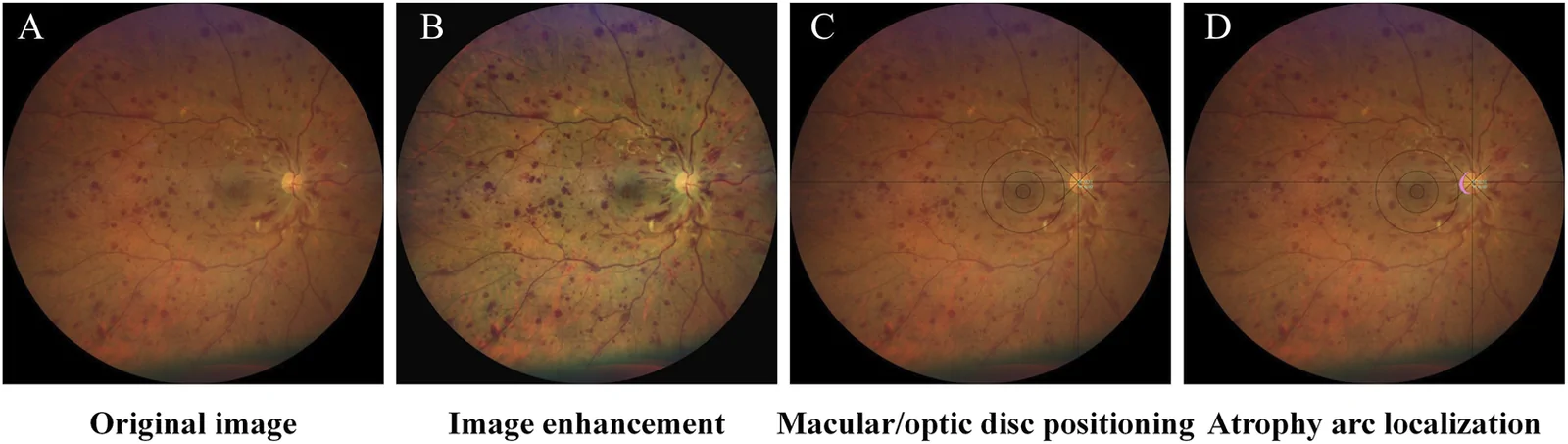
Workflow of the AI-based image analysis pipeline for RVO assessment. The schematic illustrates the key processing stages: **(A)** Input of original color fundus photographs; **(B)** Standardized image preprocessing, including quality control, color normalization, and contrast enhancement; **(C)** Localization of key anatomical structures, including the macula and optic disc; **(D)** Detection and delineation of peri-papillary atrophy (PPA) arcs.

**FIGURE 2:**
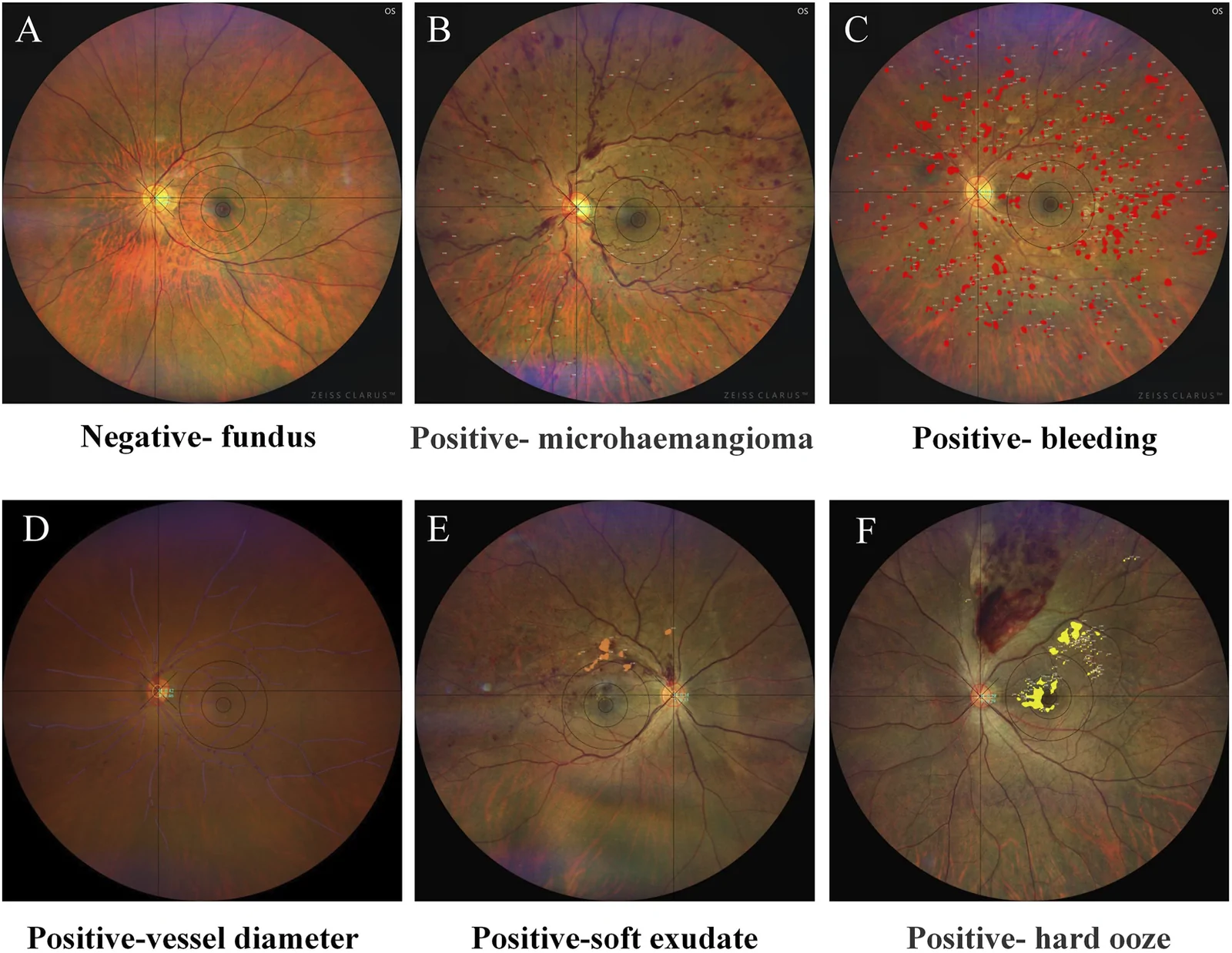
Automated annotation of RVO-specific lesions and vascular features. Comparative visualization of fundus images demonstrates the AI system’s segmentation capability: **(A)** Normal fundus without pathological findings. **(B)** Detection of microaneurysms with quantification of count and distribution. **(C)** Segmentation of hemorrhagic foci with measurement of total area and lesion size. **(D)** Vascular analysis showing diameter measurement at calibrated vessel segments. **(E)** Identification of cotton-wool spots with area quantification. **(F)** Delineation of hard exudates with precise boundary detection and area measurement.

**TABLE 2:** Quantitative analysis of vascular structure and morphology in control and RVO groups.

Fundus parameters	Control	RVO	*P* Value
Blood number	0	64.83 ± 91.15	*P* < 0.001
Blood number Distribution_Superior nasal	0	5.91 ± 13.30	*P* < 0.001
Blood number Distribution_Inferior nasal	0	7.25 ± 16.26	*P* < 0.001
Blood number Distribution_Superior temporal	0	26.57 ± 35.92	*P* < 0.001
Blood number Distribution_Inferior temporal	0	23.68 ± 38.95	*P* < 0.001
Blood area total	0	51.42 ± 88.70	*P* < 0.001
Max blood area	0	32.51 ± 63.16	*P* < 0.001
Fractal dimension	1.10 ± 0.06	1.36 ± 0.21	*P* < 0.001
Mean diameter	91.23 ± 9.48	77.84 ± 10.90	*P* < 0.001
Vessel mean curvature	0.72 ± 0.06	0.48 ± 0.38	*P* < 0.001
tRatio vessel	0.06 ± 0.01	0.04 ± 0.02	*P* < 0.001
Vessel length	1477.98 ± 214.01	50034.36 ± 112692.85	*P* = 0.028
Circle vessel fractal Z3	1.49 ± 0.03	1.33 ± 0.44	*P* = 0.005
Inferior nasal vessel fractal Z3	1.30 ± 0.05	0.89 ± 0.82	*P* < 0.001
Inferior temporal vessel fractal Z3	0.84 ± 0.08	1.05 ± 0.73	*P* < 0.001
Superior nasal vessel FractalZ3	1.30 ± 0.05	1.00 ± 0.72	*P* = 0.252
Superior temporal vessel fractal Z3	1.32 ± 0.04	0.98 ± 0.74	*P* < 0.001
Circle vessel curvature Z3	0.76 ± 0.06	0.45 ± 0.45	*P* < 0.001
Inferior nasal vessel curvature Z3	0.65 ± 0.062	0.21 ± 0.60	*P* < 0.001
Inferior temporal vessel curvature Z3	0.83 ± 0.07	0.38 ± 0.60	*P* < 0.001
Superior nasal vessel curvature Z3	0.65 ± 0.06	0.29 ± 0.54	*P* < 0.001
Superior temporal vessel curvature Z3	0.84 ± 0.08	0.40 ± 0.65	*P* < 0.001
Circle vessel diameter Z3	92.53 ± 8.67	77.95 ± 17.49	*P* < 0.001
Inferior nasal vessel diameter Z3	98.59 ± 10.60	67.10 ± 31.86	*P* < 0.001
Inferior temporal vessel diameter Z3	90.48 ± 9.17	69.16 ± 27.80	*P* < 0.001
Superior nasal vessel diameter Z3	98.59 ± 10.60	77.44 ± 30.66	*P* < 0.001
Superior temporal vessel diameter Z3	90.11 ± 8.96	71.33 ± 30.22	*P* < 0.001
Circle vessel ratio Z3	0.08 ± 0.01	0.01 ± 0.18	*P* < 0.001

### 3.3 Quantitative analysis of exudative and ischemic lesions (hard exudates and cotton-wool spots)

Macular edema is the core pathological alteration in RVO responsible for central vision loss and blurred vision (Romano et al., 2023). Hard exudates and cotton-wool spots are key fundus-visible lesions that reflect the underlying pathophysiological processes of RVO: hard exudates indicate vascular leakage, while cotton-wool spots indicate retinal microinfarction (Iijima, 2018). While definitive assessment of macular edema requires optical coherence tomography (OCT), hard exudates and cotton-wool spots, which are visible on fundus photographs, serve as surrogate indicators of exudative and ischemic pathology, respectively. Quantitative results (Table 3) showed significantly elevated metrics related to exudative pathology in RVO eyes compared to controls (*P* < 0.001). The RVO group exhibited significant increases in both the number and area of hard exudates. Similarly, the number of cotton-wool spots, their total area, and maximum area were significantly greater than in healthy eyes. These quantitative data collectively elucidate the pathophysiological changes induced by RVO, providing a critical imaging foundation for objectively assessing macular edema severity and its pathological subtypes.

**TABLE 3:** Quantitative assessment of macular edema in control and RVO groups.

Fundus parameters	Control group	RVO group	P Value
Exudate number	0	6.26 ± 12.05	*P* < 0.001
Exudate number Distribution_Superior nasal	0	0.23 ± 1.08	*P* = 0.069
Exudate number Distribution_Inferior nasal	0	0.27 ± 1.18	*P* = 0.093
Exudate number Distribution_Superior temporal	0	2.74 ± 9.19	*P* < 0.001
Exudate number Distribution_Inferior temporal	0	2.55 ± 5.125	*P* < 0.001
Exudate number Distribution_R1	0	0.22 ± 1.07	*P* = 0.273
Exudate number Distribution_R2	0	0.94 ± 2.62	*P* = 0.003
Exudate number Distribution_R3	0	2.67 ± 5.78	*P* < 0.001
Exudate number Distribution_R3_	0	3.04 ± 7.55	*P* < 0.001
Exudate area total	0	0.42 ± 1.16	*P* < 0.001
Max exudate area	0	0.26 ± 0.82	*P* < 0.001
Soft exudate number	0	2.10 ± 2.81	*P* < 0.001
Soft exudate number Distribution_Superior nasal	0	0.27 ± 1.04	*P* = 0.008
Soft exudate number Distribution_Inferior nasal	0	0.07 ± 0.32	*P* = 0.126
Soft exudate number Distribution_Superior temporal	0	1.08 ± 2.06	*P* < 0.001
Soft exudate number Distribution_Inferior temporal	0	0.68 ± 1.43	*P* < 0.001
Soft exudate number Distribution_R1	0	0	*P* = 0.085
Soft exudate number Distribution_R2	0	0.05 ± 0.05	*P* = 0.931
Soft exudate number Distribution_R3	0	0.46 ± 1.17	*P* = 0.005
Soft exudate number Distribution_R3_	0	1.4 ± 2.44	*P* < 0.001
Soft exudate area total	0	0.55 ± 1.41	*P* < 0.001
Max soft exudate area	0	0.28 ± 0.58	*P* < 0.001

### 3.4 Quantitative analysis of FTD

Fundus tessellation (FT), also known as tessellated fundus, is a common fundus manifestation characterized by increased visibility of the underlying large choroidal vessels due to reduced pigmentation in the retinal pigment epithelium (RPE) and choroid. Previous research has identified a significant association between RVO and more pronounced peripapillary fundus tessellation progression (*P* < 0.001) (Yan et al., 2018). However, traditional assessment of FT relies primarily on subjective clinician grading, lacking objective, reproducible quantification standards. In this study, we applied an automated image quantitative analysis system to evaluate fundus FTD in both RVO and control groups. As visually presented in Figure 3, representative fundus images and their corresponding pseudo-color density maps illustrate a consistent trend of increased FTD visibility in RVO patients compared to controls. Quantitative analysis further confirmed a consistent trend of higher FTD values in the RVO group across all assessed regions, including the peripapillary area, macular area, and the combined papillo-macular region, despite these differences not reaching statistical significance (all *P* > 0.05; Table 4).

**FIGURE 3:**
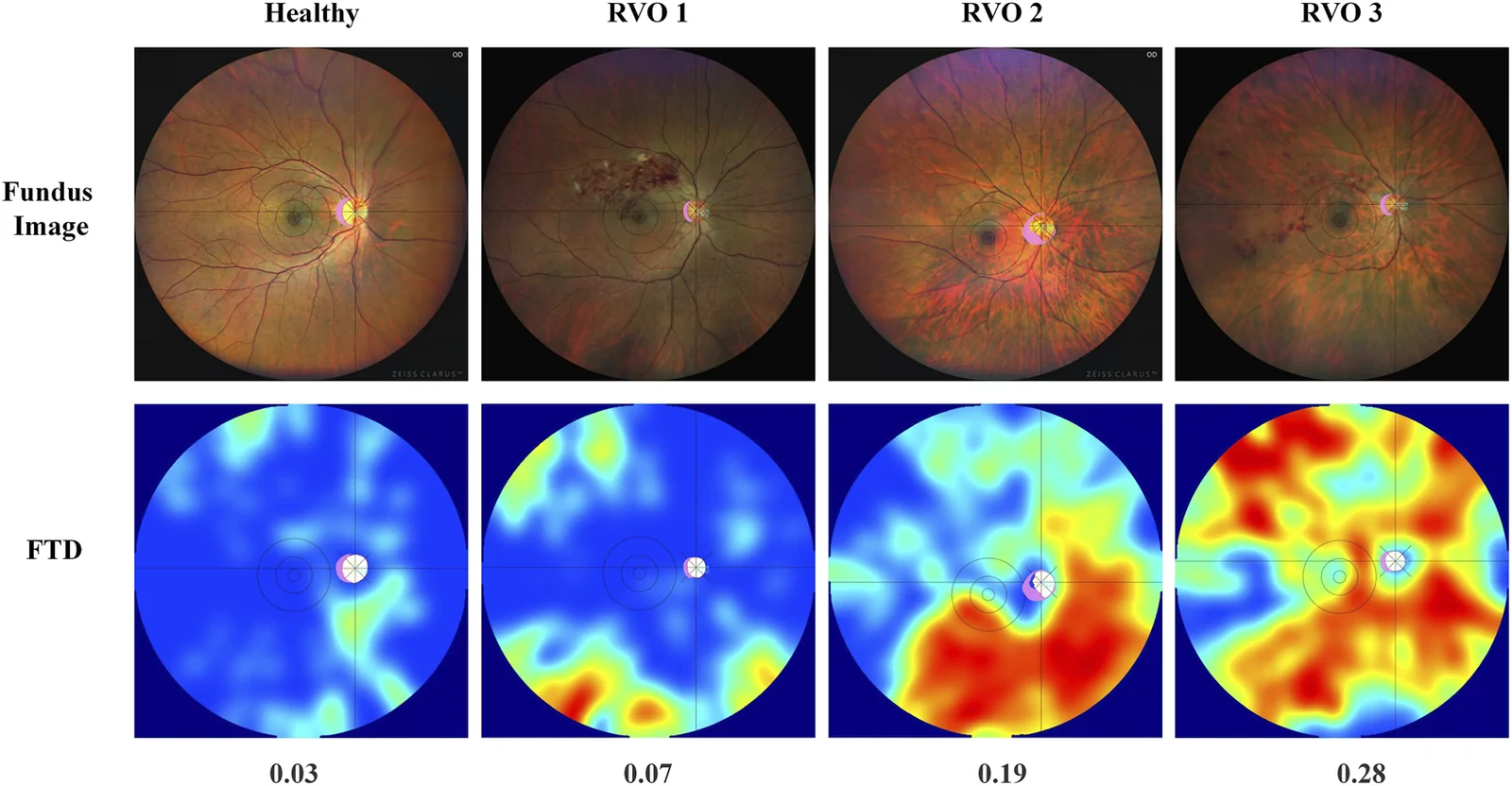
Quantitative Analysis of FTD in Control and RVO Groups. Representative color fundus photographs (upper panel) and their corresponding pseudo-color tessellation density maps (lower panel) are displayed, in which the color gradient from blue to red indicates increasing tessellation density. Quantitative analysis showed that, although FTD values were numerically higher in the RVO group, the differences did not reach statistical significance compared with the control group (all *P* > 0.05).

**TABLE 4:** Quantitative analysis of FTD in control and RVO groups.

Fundus parameters	Control group	RVO group	*P* Value
Overall FTD	0.095 ± 0.056	0.11 ± 0.067	*P* = 0.466
Optic disc FTD	0.048 ± 0.063	0.053 ± 0.058	*P* = 0.375
Macular FTD	0.029 ± 0.047	0.048 ± 0.086	*P* = 0.633
Disc-macula combined FTD	0.041 ± 0.056	0.063 ± 0.077	*P* = 0.133

### 3.5 Correlation analysis of quantitative parameters with RVO disease status

Finally, we assessed the association between quantitative imaging parameters and RVO status using univariate GEE analysis. As shown in Table 5, a higher number of hemorrhages, increased hard exudates, elevated Fractal Dimension, hypertension, and dyslipidemia were significantly associated with RVO, whereas decreased tRatio Vessel and narrowed Circle Vessel Diameter Z3 were significantly associated with increased RVO. The corresponding unadjusted odds ratios and 95% confidence intervals are presented in Table 5. Figure 4 further visualizes the distribution differences of these key vascular parameters (Fractal Dimension, tRatio Vessel, and Circle Vessel Diameter Z3) between the RVO and control groups using violin plots, complementing these findings. Collectively, these results demonstrate that the multi-dimensional quantitative metrics extracted by our system hold significant value for RVO characterization and pathophysiological mechanism analysis.

**TABLE 5:** Univariate and multivariable associations of quantitative imaging parameters with RVO status.

Variable	Unadjusted OR (95% CI)	P Value	Adjusted OR (95% CI)	P Value
Lesion burden parameters				
Blood number	NE†	<0.001	—	—
Exudate number	NE†	<0.001	—	—
Soft exudate number	NE†	<0.001	—	—
Vascular morphological parameters				
Fractal dimension (per 0.1 increase)	1.96 (1.19, 3.21)	0.008	3.14 (1.75, 5.62)	<0.001
tRatio vessel (per 1% increase)	0.48 (0.35, 0.66)	<0.001	0.52 (0.38, 0.71)	<0.001
Circle vessel diameter Z3 (per 1 μm increase)	0.89 (0.84, 0.95)	<0.001	0.928 (0.812,1.061)	<0.001
Systemic risk factors				
Hypertension	16.51(7.12, 38.31)	<0.001	3.21 (1.54, 6.70)	0.002
Dyslipidemia	4.02 (1.98, 8.16)	<0.001	2.45 (1.23, 4.88)	0.011
Demographic factors				
Age (per 1 year)	—	—	1.08 (1.01, 1.15)	0.021
Sex (male vs. female)	—	—	NS	0.908
Eye side (right vs. left)	—	—	NS	0.422

†NE, not estimable. Due to complete separation (all control eyes had zero counts for hemorrhages, hard exudates, and soft exudates), the OR, did not converge to a finite estimate. Fisher’s exact test was therefore used to provide reliable P values. Dashes (−) indicate variables not included in the corresponding model. Multivariable model included age, sex, eye side, Fractal Dimension, tRatio Vessel, hypertension, and dyslipidemia, with GEE, and cluster-robust standard errors to account for inter-eye correlation. NS, not significant.

**FIGURE 4:**
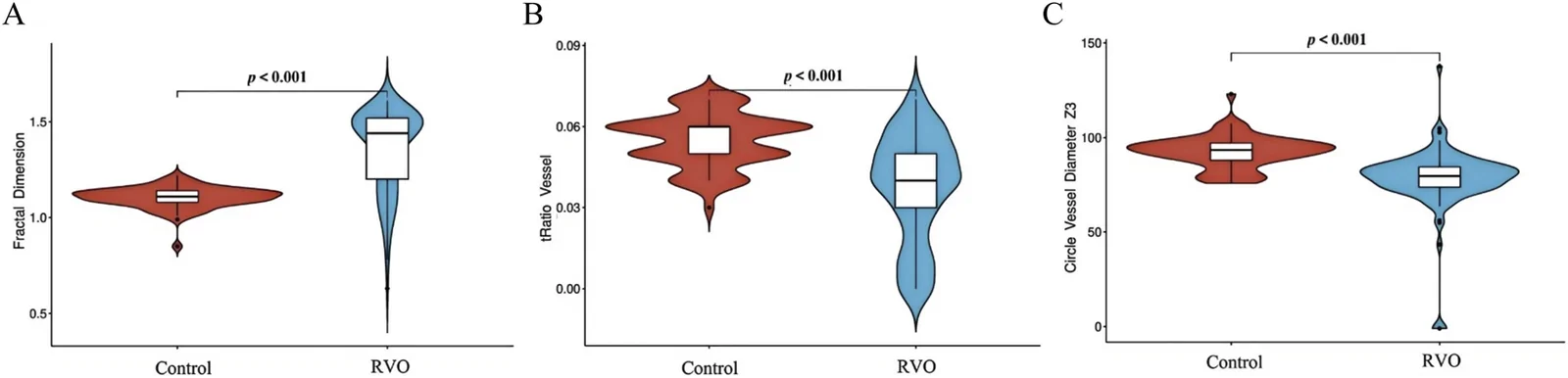
Comparative analysis of key quantitative indicators between control and RVO groups using violin plots. The distributions of the following parameters are shown: **(A)** Fractal dimension, **(B)** tRatio Vessel (vessel density), and **(C)** Circle Vessel Diameter Z3 (mean vessel diameter within a 13.5 mm circle). Statistical analysis using the Mann–Whitney U test revealed significant differences in all metrics between the RVO group and the control group (all *P* < 0.001).

To identify independent correlates of RVO while adjusting for potential confounders, we performed a multivariable logistic regression analysis using GEE with cluster-robust standard errors to account for inter-eye correlation. The model included age, sex, eye side, Fractal Dimension, tRatio Vessel, Circle Vessel Diameter Z3, hypertension, and dyslipidemia, with hypertension and dyslipidemia being significantly imbalanced between groups at baseline. In the multivariable analysis, each 0.1-unit increase in Fractal Dimension was independently associated with a significantly elevated odds of RVO (adjusted OR = 3.14; 95% CI: 1.75–5.62; P < 0.001), whereas each 1% increase in tRatio Vessel was independently associated with a reduced odds of RVO (adjusted OR = 0.52; 95% CI: 0.38–0.71; P < 0.001). Circle Vessel Diameter Z3 (adjusted OR = 0.928; 95% CI: 0.812–1.061; P < 0.001) was also independently associated with RVO. Hypertension (adjusted OR = 3.21; 95% CI: 1.54–6.70; P = 0.002) and dyslipidemia (adjusted OR = 2.45; 95% CI: 1.23–4.88; P = 0.011) were also independently associated with RVO. Age was identified as an independent correlate, with each additional year associated with an 8% increase in odds (adjusted OR = 1.08; 95% CI: 1.01–1.15; P = 0.021). Sex and eye side were not significantly associated with RVO (P = 0.908 and P = 0.422, respectively). Collectively, these multivariable findings confirm that higher Fractal Dimension, lower tRatio Vessel, narrowed Circle Vessel Diameter Z3, hypertension, dyslipidemia, and older age are independently associated with RVO, further supporting the pathophysiological relevance of vascular structural and morphological alterations.

### 3.6 Model performance evaluation

To evaluate the diagnostic capability of the AI system for RVO identification, we constructed a classification model based on the multi-dimensional quantitative parameters extracted by the system (including lesion metrics, vascular morphological features, and FTD). Using 5-fold cross-validation to avoid overestimation, the model’s performance was assessed on the entire dataset of 166 eyes (106 RVO, 60 controls). As presented in Table 6, the cross-validated mean accuracy was 94.8% (SD: 1.2%; range: 93.1%–96.4%), sensitivity 94.9% (SD: 1.5%; range: 92.5%–96.8%), specificity 94.5% (SD: 1.8%; range: 91.6%–96.2%), and the mean AUC was 0.951 (SD: 0.018; range: 0.932–0.965). It should be noted that this AUC was derived from internal 5-fold cross-validation on a single-center dataset and represents an estimate of internal validity rather than a guarantee of generalizable real-world performance. These results indicate that the AI system has excellent and stable discriminative power for distinguishing RVO eyes from healthy controls in this internal validation setting.

**TABLE 6:** Cross-validated diagnostic performance of the RVO AI model.

Metric	Cross-validated mean (SD)	Range across folds
Accuracy	94.8% (1.2%)	93.1%–96.4%
Sensitivity	94.9% (1.5%)	92.5%–96.8%
Specificity	94.5% (1.8%)	91.6%–96.2%
AUC	0.951 (0.018)	0.932–0.965

## 4 Discussion

This study successfully applied an automated image quantitative analysis system to perform precise lesion localization and multi-dimensional analysis in a cohort of 166 eyes. The results validate the feasibility of this technology for identifying and quantifying key RVO lesion characteristics, thereby demonstrating strong potential for developing a fully automated, image-based segmentation and assessment system for RVO.

Our findings align with the broader trajectory of AI application in RVO diagnosis. Recent years have witnessed significant progress in this domain, particularly with the development and validation of various deep learning models (Chen et al., 2021; Ji et al., 2024; Beeche et al., 2023). For instance, Swin Transformer architectures have been employed to classify RVO into central CRVO, BRVO, and macular (MRVO) types, outperforming traditional methods (Ji et al., 2024). Another study proposed a Neural Understanding Network (NUN) that combines convolutional and graph neural networks, significantly enhancing RVO classification accuracy (Beeche et al., 2023). Chen et al. also demonstrated that deep learning models can assist in RVO diagnosis using color fundus photographs, achieving high sensitivity and specificity (Chen et al., 2021). However, most existing AI models for RVO focus primarily on binary or multi-class classification and do not provide quantitative assessment of lesion burden or vascular morphology, which are essential for objective severity grading and longitudinal monitoring. In contrast, our system offers a more comprehensive evaluation by integrating pixel-wise lesion segmentation with quantitative vascular parameter extraction, including fractal dimension, vessel density, and vessel curvature. This integrated approach provides clinicians with objective and multidimensional information beyond simple classification, potentially facilitating disease severity stratification, treatment response monitoring, and personalized management of RVO patients.

This research confirms the technical feasibility of automated RVO lesion segmentation using fundus images. The system is capable of accurately identifying characteristic fundus manifestations of RVO, including dilated retinal veins, intraretinal and subretinal hemorrhages, macular edema, hard exudates, and cotton-wool spots. (Zhang et al., 2022; Chen et al., 2024). In vascular morphology analysis, our system accurately identified key differences between RVO and healthy eyes: significant increases in the number and area of hemorrhages, elevated vascular fractal dimension, and narrowed mean vessel diameter coupled with reduced vessel density. These quantitative features align with conclusions from previous RVO pathophysiological studies (Long et al., 2022; Koulisis et al., 2017), thereby validating the system’s reliability. Crucially, our study achieved efficient and precise extraction of these multi-dimensional features through automated image processing, robustly demonstrating the stability and repeatability of automated lesion segmentation.

Beyond standard color fundus photographs, the application of ultra-widefield (UWF) imaging in AI-assisted RVO diagnosis has shown promise in improving detection sensitivity by covering broader retinal areas (Wu T. et al., 2024; Nagasato et al., 2018). DL models applied to UWF images have demonstrated superior performance in detecting CRVO and BRVO compared to traditional machine learning methods like SVM (Nagasato et al., 2018; Nagasato et al., 2019; Liu et al., 2025). While our current system utilizes images acquired with an ultra-widefield camera (Zeiss CLARUS 500), the analysis and showcased results focus on the macular-centered region. This approach ensures consistency with the vast majority of clinical studies using standard field fundus photos and directly targets the region most critical for RVO visual prognosis. The use of UWF imaging provides a high-quality data source, and future work will leverage its full field to analyze peripheral pathologies.

Automated identification of ischemic burden represents a critical advancement in AI-assisted RVO assessment. A critical advancement in AI for RVO is the automated analysis of ischemic areas and non-perfusion areas (NPA) from color fundus photographs, potentially avoiding the need for invasive fluorescein angiography (FFA) (Miao et al., 2022; Masayoshi et al., 2024). Studies have successfully trained models to identify NPA in BRVO patients based solely on CFPs (Miao et al., 2022). Although our system does not directly segment NPA, its quantitative assessment of hemorrhages and microaneurysms indirectly reflects ischemic burden, and future integration of explicit NPA segmentation could further augment its prognostic utility. Furthermore, our system successfully differentiated between hard exudates and cotton-wool spots on fundus photographs, enabling precise quantification of their number and spatial distribution. Hard exudates originate from the leakage of lipids or lipoproteins from plasma through retinal vessels, indicating active vascular leakage, whereas cotton-wool spots arise from microinfarctions in the retinal nerve fiber layer, signifying retinal microcirculatory ischemia (Romano et al., 2023; Iijima, 2018). The accurate distinction and precise quantification of these features are crucial for guiding subsequent RVO treatment and prognostic assessment. This capability is consistent with efforts to combine multimodal images (e.g., CFPs and FFA) using meta-learning approaches for RVO detection with limited data (Jiachu et al., 2024), as well as with AI systems designed to interpret FFA angiograms rapidly and consistently (Huang et al., 2024).

The clinical relevance of AI-derived quantitative parameters is underscored by studies exploring the relationship between RVO hemorrhage patterns (e.g., depth in CRVO) and visual outcomes (Au et al., 2020), as well as investigations linking retinal vascular geometric features (e.g., arteriovenous crossings) to RVO development (Muraoka and Tsujikawa, 2019; Choi et al., 2025). Our correlation analysis reinforces this, showing strong associations between hemorrhage count, exudate number, vascular fractal dimension, vessel density, diameter, and RVO disease status. These multi-dimensional metrics extracted by our system show significant value for characterizing RVO and suggest potential as imaging biomarkers, pending further validation against other retinal diseases and pathophysiological mechanism analysis.

This study also innovatively incorporated FTD, an indicator of choroidal status, into the RVO evaluation framework. We found a consistent, though statistically non-significant, trend of increased FTD in key regions like the peripapillary and macular areas in the RVO group. This phenomenon may be associated with pathological processes such as altered choroidal hemodynamics and choroidal thinning subsequent to RVO (Zhang et al., 2022; Yan et al., 2018). This implies that FTD could potentially serve as an important bio-imaging marker for auxiliary diagnosis, risk stratification, and prognosis assessment in RVO, offering a new quantitative dimension. This approach resonates with research utilizing AI for quantitative assessment of fundus tessellation in other chorioretinal diseases (Muraoka and Tsujikawa, 2019; Deng et al., 2025).

While this study demonstrates the technical feasibility and clinical potential of our approach, we acknowledge several limitations. First, this is a single-center study with a relatively modest sample size, lacking external validation. Second, and most importantly, the study only distinguished RVO from healthy controls; we did not include other retinal diseases (e.g., diabetic retinopathy, age-related macular degeneration) as comparators. As hemorrhages and exudates are hallmark features of RVO, the diagnostic task in this study was inherently less complex than real-world differential diagnosis, and the reported specificity may be overestimated in the absence of disease controls. This concern is well recognized in the AI ophthalmology literature: case-control designs that select participants at the extremes of the target condition carry a high risk of overestimating test performance (Kang et al., 2024), and models trained on curated datasets often show significant performance drops when applied to more diverse real-world populations (Pourjavan et al., 2025). Third, although we used 5-fold cross-validation to mitigate overfitting, an independent external test set is still required to confirm generalizability. It should be noted that the performance reported in this study was derived from internal cross-validation on a single-center dataset. This does not substitute for true external validation on an independent dataset from a different institution or population. The lack of an external test set remains a major limitation, as model performance may vary considerably across different fundus cameras, imaging protocols, and patient demographics. Therefore, the reported AUC of 0.951 should be interpreted as an estimate of internal validity rather than a guarantee of generalizable real-world performance. Fourth, the cross-sectional design precludes causal inference, and effect estimates should therefore be interpreted as associations rather than causal effects.

From a clinical perspective, the proposed system has several potential applications. First, it could serve as a screening tool in primary care or community settings, where non-ophthalmic specialists may lack the expertise to detect early RVO lesions (Gu et al., 2024). The automated segmentation of hemorrhages, hard exudates, and cotton-wool spots could help reduce missed diagnoses and facilitate timely referral. Second, the quantitative metrics provided by the system, such as lesion area, vessel density, and fractal dimension, could assist clinicians in objectively monitoring disease progression and treatment response, particularly in patients receiving anti-VEGF therapy (Li et al., 2026). Third, the system’s ability to quantify vascular morphological changes may offer insights into RVO pathophysiology and support individualized risk stratification (Torres-Costa et al., 2026). However, it is important to emphasize that these applications remain theoretical until the system undergoes rigorous prospective validation in real-world clinical settings.

Future directions should involve multi-center, large-scale prospective studies with diverse disease controls to further validate the system’s generalizability and clinical utility (Lendzioszek et al., 2024; Ji et al., 2024; Nilforoushan et al., 2026). Validation in cohorts encompassing other retinal diseases and mild or early RVO cases would better reflect real-world clinical performance and is essential to establish the system’s diagnostic specificity. Integration with advanced imaging modalities such as optical coherence tomography angiography (OCTA) and FFA could provide deeper insights into perfusion characteristics and strengthen the system’s diagnostic and prognostic capabilities (Long et al., 2022; Anzidei et al., 2022). Ultimately, these advancements hold the potential to transform the traditional subjective diagnostic paradigm for RVO, steering clinical workflows toward standardization, precision, and intelligence.

## 5 Conclusion

In summary, the present study demonstrates the feasibility of an AI-based automated segmentation and quantitative analysis system for RVO lesions on color fundus photographs. Our findings indicate that the system enables precise lesion segmentation and quantitative metric extraction, and that AI-derived vascular parameters, particularly Fractal Dimension and tRatio Vessel, are independently associated with RVO after adjustment for demographic factors and systemic vascular risk factors. These observations offer preliminary evidence supporting the potential utility of this system for objective RVO assessment. Nevertheless, given the single-center cross-sectional design, the absence of external validation, and the lack of disease controls (e.g., diabetic retinopathy, age-related macular degeneration), the present results must be interpreted with due caution. Rigorous prospective multi-center studies incorporating external validation and diverse disease controls are essential before this system can be integrated into routine clinical practice.

## Data Availability

The original contributions presented in the study are included in the article/supplementary material, further inquiries can be directed to the corresponding authors.
